# Mechanistic insights derived from re-establishment of desiccation tolerance in germinating xerophytic seeds: *Caragana korshinskii* as an example

**DOI:** 10.3389/fpls.2022.1029997

**Published:** 2022-11-07

**Authors:** Long Peng, Xu Huang, Manyao Qi, Hugh W. Pritchard, Hua Xue

**Affiliations:** ^1^ The Research Institute of Subtropical Forestry, Chinese Academy of Forestry, Hangzhou, China; ^2^ National Engineering Research Center of Tree breeding and Ecological remediation, College of Biological Sciences and Biotechnology, Beijing Forestry University, Beijing, China; ^3^ Chinese Academy of Sciences, Kunming Institute of Botany, Kunming, China; ^4^ Royal Botanic Gardens, Kew, Wakehurst, West Sussex, United Kingdom

**Keywords:** *caragana korshinskii*, desiccation tolerance, germplasm conservation, seed, xerophytic woody species

## Abstract

Germplasm conservation strongly depends on the desiccation tolerance (DT) of seeds. Xerophytic seeds have strong desiccation resistance, which makes them excellent models to study DT. Although some experimental strategies have been applied previously, most methods are difficult to apply to xerophytic seeds. In this review, we attempted to synthesize current strategies for the study of seed DT and provide an in-depth look at *Caragana korshinskii* as an example. First, we analyze congenital advantages of xerophytes in the study of seed DT. Second, we summarize several strategies used to study DT and illustrate a suitable strategy for xerophytic species. Then, based on our previous studies work with *C. korshinskii*, a feasible technical strategy for DT re-establishment is provided and we provide illustrate some special molecular mechanisms seen in xerophytic seeds. Finally, several steps to unveil the DT mechanism of xerophytic seeds are suggested, and three scientific questions that the field should consider are listed. We hope to optimize and utilize this strategy for more xerophytic species to more systematically decipher the physiological and molecular processes of seed DT and provide more candidate genes for molecular breeding.

## Introduction

The safe conservation of plant genetic resources is essential for breeding programs and is an underpinning technology to guarantee adequate food supplies for future human generations (Villalobos and Engelmann, 1995; Gonçalves et al., 2019). Such conservation, with the aim of sustainable utilization, can be realized through several forms of germplasm, such as seeds, living plants in germplasm nurseries, and plant tissue *in vitro* (Dinato et al., 2020). Among these, seed storage is the most effective and cost-efficient means of long-term *ex-situ* preservation of genetic diversity for both crops and wild plant diversity (Chapman et al., 2018; Wade et al., 2020).

Large-scale *ex-situ* seed banking was first implemented in the early 20^th^ century to maintain and protect crop lines and over the last 50 years has been increasingly used to preserve wild plants diversity too (Chapman et al., 2018; Wade et al., 2020). As back-up sites for the global conservation system, seed vaults complement *in situ* conservation and play a functional and symbolic role for enhanced integration and cooperation to conserve crop and wild plant diversity (Li and Pritchard, 2009; Westengen et al., 2013). Hitherto, almost 57,000 taxa are preserved in more than 350 institutions around the world (Chapman et al., 2018), such as the Svalbard Global Seed Vault for crops in Norway (Asdal and Guarino, 2018), the Royal Botanic Gardens Kew’s Millennium Seed Bank for wild species in the United Kingdom (Liu et al., 2018), the National Plant Germplasm System for crops in the United States of America (Byrne et al., 2018), and the Germplasm Bank of Wild Species (GBoWS) in China (Li et al., 2010).

A critical early step in seed banking is drying the material to a moisture content low enough to avoid ice formation on storage at sub-zero temperatures. However, different species produce seeds with varying levels of desiccation tolerance (DT). When considering DT, seeds are divided into orthodox, intermediate and recalcitrant (Roberts, 1973; Ellis et al., 1990; Ballesteros et al., 2021). Orthodox seeds, include those of many crops, can tolerate desiccation to 3-7% moisture content and potentially survive *ex situ* storage for many years under conventional gene-bank conditions of -20°C (Stanwood, 1989; Sara et al., 2014; Colville and Pritchard, 2019). However, about 8% of the world’s flowering plants are estimate to have seeds that are intolerant to drying (Wyse and Dickie, 2017). Such recalcitrant seeds are often sensitive to drying below *c.* 35% moisture content and tend to be a feature of wet forest species, including cocoa, coconut, rubber tree, oaks and dipterocarps (Pammenter and Berjak, 1999; Black and Pritchard, 2002). In contrast, intermediate seeds have partial DT (*e.g*., to *c.* 10% moisture content) and may undergo a precipitous loss of viability when stored at -20°C. Examples include economically valuable species such as tea, oil palm, citrus and coffee (Dussert et al., 2018). This interplay between drying level and storage temperature applies beyond intermediate seeds. For example, desiccation and cryopreservation (often in liquid nitrogen at -196°C) is an applicable technology for germplasm conservation as long as the seeds are not ultradry, as deep cooling can then lead to structural instability (Li and Pritchard, 2009; Ballesteros et al., 2021). Alternatively, there is interest in storing seeds ultra-dry without cooling as this can reduce the cost of seed storage, *e.g.*, in the tropics (Zheng et al., 1998), Thus seed DT is a complex function of the level of water loss, the kinetics of dehydration, the drying time duration and any interaction with storage temperature.

More generally in plant sciences, DT is widely defined as the ability of an organism to withstand or endure extreme dryness or dehydration-like conditions (Black and Pritchard, 2002; Oliver et al., 2020), and unveiling the molecular mechanism of DT offers the prospect by genetic engineering of identifying and using novel DT genes for crop improvement and enhancing the storability of recalcitrant seeded species.

Xerophytic seeds can germinate in arid and semi-arid regions, and even in deserts. They must evolutionarily develop resistance mechanisms to adapt or tolerate stress to survive in dry, sandy environments (Gong et al., 2015). Hence, they logically provide a potentially excellent experimental material to explore DT mechanisms. However, due to sampling collection difficulties, which required long experimental periods, and other reasons, these species have been mainly overlooked. Consequently, such mechanisms of stress tolerance and resilience during the early stage of germination in xerophytic seed plants are primarily uncharted.

This review article synthesizes current evidence in order to decipher the potential DT mechanisms of dry plant seeds. Firstly, we describe the favorable aspects of xerophytic species’ seeds in DT studies, particularly in relation to the re-establishment of DT in germinating seeds. After reviewing recent progress in this research area, we take *Caragana korshinskii* seeds as a specific example of the functional response to the re-establishment of DT. This strategy may be suitable for exploring the mechanisms of DT in xerophytic seeds.

## Subsections relevant for the subject

### Congenital advantages of xerophytes in the study of seed DT

Dryland ecosystems are ecologically distinct, increasing in global extent under shifting climates and have been recognized as degraded in over 50% of their range (Shackelford et al., 2021). Various ecosystem assessments have indicated this is a threatened habitat and intervention is needed (Louhaichi et al., 2014; Pérez et al., 2019; Miguel et al., 2020; Shackelford et al., 2021). For this, it is best to use native species, particularly shrubby xerophytes. In the drylands of northwestern China, several drought‐resistant plant species have been widely planted since the 1970s (Yu and Wang, 2018). The selected species are pioneer xerophytic plants, such as *Haloxylon ammodendron* (Fan L et al., 2018), *Hedysarum scoparium* (Su et al., 2016), *Hippophae rhamnoides* (Ghangal et al., 2012), *Zygophyllum xanthoxylon* (Shi and Zhang, 2015), *Calligonum mongolicum* (Fan B et al., 2018), and *Caragana korshinskii* (Yang et al., 2014). They have broad functionality relating to soil and water conservation, desertification prevention and control, and vegetation restoration in arid and semiarid regions (Yu and Wang, 2018). Xerophytic plants are often exposed to stressful conditions, such as water deficit, dry air, high irradiance, and extreme temperatures, including during the germination phase of the life cycle.

With the development of molecular biology and omics, *i.e.*, transcriptome, and proteome and metabolome, drought-resistance in some model plants and crops has already been thoroughly elucidated (Zhu, 2016; Qi et al., 2018; Gong et al., 2020). Adopting these technical strategies, we can decipher the mechanism of DT of xerophytic plant seeds and identify high-quality candidate genes for improving DT. Opportunities to do this are greatest with representatives of the families for which there are the most published plant genomes, and the top three families are Poaceae, Brassicaceae, and Fabaceae (Sun et al., 2022). *Caragana korshinskii* is a member of the Fabaceae for which there have been around 50 sequenced genomes (Sun et al., 2022). Furthermore, several seeds of species in Fabaceae relate to the study of seed DT like *Pisum sativum*, *Sesbania virgate*, *Medicago truncatula*, and V*igna unguiculata* (Buitink et al., 2003; Leprince et al., 2004; Faria et al., 2005; Boudet et al., 2006; Buitink et al., 2006; Terrasson et al., 2013; Masetto et al, 2014; Masetto et al, 2016; Silva et al., 2017; Wang et al., 2021). Among them, DT in seeds of *M. truncatula* is well studied (Buitink et al., 2003; Leprince et al., 2004; Faria et al., 2005; Boudet et al., 2006; Buitink et al., 2006; Terrasson et al., 2013). These all position Caragana as the species complementary to Medicago.

### Review of research strategies used by DT studies

**Figure 1:**
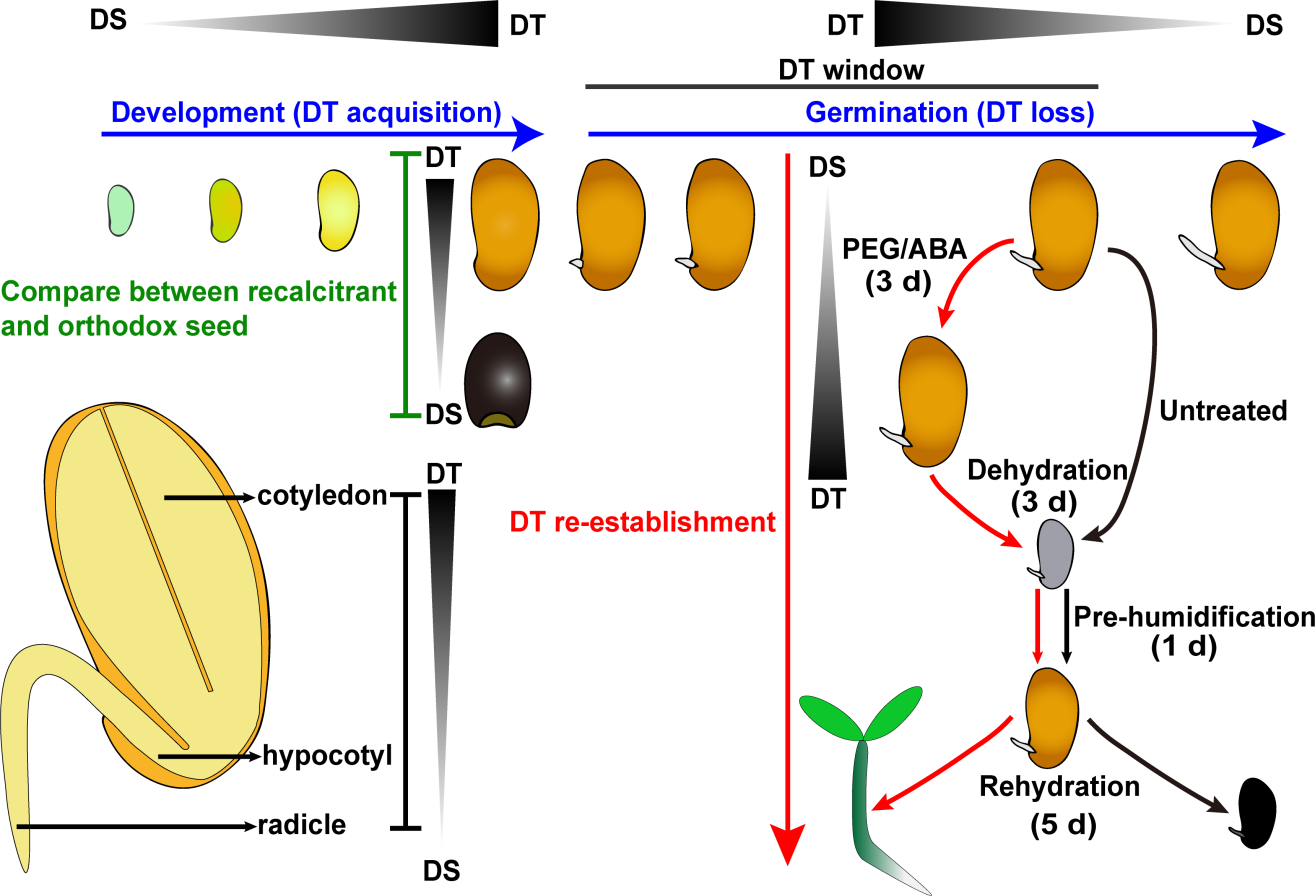
Schematic summary of the strategies used within DT studies in seed. Comparative analysis between orthodox and recalcitrant seeds is a handy strategy for depicting the character of DT (green words). Moreover, DT acquisition and loss accompanies the physiological processes of seed development and germination, respectively (blue words). When germinating seeds at the stage of radicle protrusion are fast dried (black arrows, 3 d), they do not survive, as shown by the lack of seedling growth after pre-humidification and rehydration. If drying treatment is preceded by PEG/ABA treatment (red arrows), after pre-humidification, seeds survive, as shown by further growth and seedling establishment. Emphasizing a PEG/ABA treatment can re-establish tolerance against desiccation treatment (red words). The detailed process of DT re-establishment of *C. korshinskii* seed is as follows: Seeds are germinated for 12 h, and then dried either directly or after 3 d of incubation in PEG 6000 solution of –1.7 Mpa at 10°C. After incubation, germinating seeds are rinsed thoroughly in distilled water and then dehydrated in silica gel for 3 d, pre-humidified for 1 d, and imbibed for 5 d. The total duration of the experiment is about 2 weeks. After PEG treatment, 81% of radicles recovered DT, which means that *C. korshinskii* seed radicle successfully re-establish DT (Peng et al., 2017). In addition, the radicles appear to be the most desiccation-sensitive part of germinating seeds, followed by the hypocotyls and cotyledons (the lower left part). A triangle with a gradient of black and grey indicates the DT transition. DT, desiccation tolerance; DS, desiccation-sensitive.

According to genetic background of species, and the physiological change during seed development and germination, some strategies have been developed in previous studies to increase understanding of the mechanism(s) of seed DT and simultaneously have screened potential DT relative genes (**Figure 1**). Here, we introduce the principles and scope of application of these strategies.

#### Comparing orthodox and recalcitrant seeds

Unlike orthodox types, recalcitrant seeds are highly sensitive to desiccation (Pammenter and Berjak, 1999; Dussert et al., 2018). On account of the DT differences, comparative proteomic and transcriptomic analysis between orthodox and recalcitrant seeds has proved to be a useful strategy for depicting the character of DT (Pukacka and Ratajczak, 2007; Delahaie et al., 2013; Ratajczak et al., 2013). The con-generic species Norway maple (*Acer platanoides*) and sycamore (*A. pseudoplatanus*), which can grow under similar climatic conditions, produce seeds with different tolerances to desiccation (Pukacka and Ratajczak, 2007). In these two species, changes in physiological or epigenetic processes mirror the response of the seeds to severe desiccation, such as changes in the antioxidant system and in the genomic 5-methylcytosine level (Pukacka et al., 2011; Ratajczak et al., 2013; Plitta-Michalak et al., 2018; Stolarska et al., 2020; Wojciechowska et al., 2020).

Inter-species comparison of seeds with different levels of DT at the point of natural dispersal provides a direct way of screening the frequency of DT in the plant kingdom (Pritchard et al., 2004). However, such an approach on species with diverse genetic background and phenotypic plasticity may be impacted by seed provenance and the maternal environment. For example, DT level varies significantly in seed lots of the recalcitrant-seeded species *Aesculus hippocastanum* when collected from sites across Europe (Daws et al., 2010). Thus, without knowing the precise stage of development in two species, inferences about physiological processes may be misleading and irrelevant genes inadvertently revealed (**Table 1**). Even if the genotype variation within the same genus is reduced to a minimum, *i.e.* relatedness is maximised, species-specific genes are still not guaranteed to be associated with DT. Thus, due to species-specific influences, comparing differences between recalcitrant and orthodox seeds cannot accurately elucidate the mechanism of DT (Lang et al., 2014).

**Table 1:** Principle, advantages, and disadvantages of strategies for studying the mechanism of DT in seeds.

Strategies	Principle	Advantages	Disadvantages
The comparison of distinct DT at seed dispersal	Different DT occurred in orthodox and recalcitrant seeds.	(1) Simple operation (2) Short experimental period	Species-specific influence
The acquisition of DT in developing orthodox seeds	Orthodox seeds acquired DT gradually during seed development.	(1) Unchanged genetic background (2) Decipher the DT mechanisms directly	(1) Long experimental period (2) Seed developmental interference (3) Continuous sample collection
The loss of DT during germination	Seeds lost DT progressively during seed germination.	(1) Avoidance of the disadvantage in continuous sample collection (2) Short experimental period (3) Unchanged genetic background	(1) Seed germinative interference (2) Indirect reflection of the mechanism of DT in seed
The re-establishment of DT in post-germinating seeds	PEG and/or ABA can rescue DT during seed germination.	(1) Simple operation (2) Short experimental period (3) Unchanged genetic background (4) Avoidance of the disadvantage in continuous sample collection (5) Avoidance of the seed developmental and germinative interference (6) Decipher the DT mechanisms directly	Artificial, more experimental factors involved

#### DT acquisition during orthodox seed maturation

During the development of orthodox seeds, water loss occurs gradually near the end of the accumulation of storage compounds, while within a similar time window, DT is acquired gradually (Li et al., 2011; Huang and Song, 2013; Lang et al., 2014; Peng et al., 2021). The transition of developing seeds from the phase of reserve accumulation to maturation drying is associated with distinct gene expression and metabolic switches, which are strongly coupled with longevity and impact seed storage (Fait et al., 2006; Angelovici et al., 2010; Chatelain et al., 2012; Verdier et al., 2013). In this phase, the synthesis of storage and protection proteins, such as late embryogenesis abundant (LEA) proteins, heat shock proteins (HSPs), and proteins involved in stress defense (antioxidant system and secondary metabolites), serve as shields to prevent cellular collapse during low water activities (Tunnacliffe and Wise, 2007; Huang et al., 2012; Almoguera et al., 2015; Lang et al., 2017).

Non-reducing sugars, such as sucrose and oligosaccharides are crucial protective compounds in the response of seeds to desiccation stress. They play a prominent role in protecting the tissues *via* water replacement and vitrification during seed dehydration (Black et al., 1999; Li et al., 2011; Lang et al., 2017; Leprince et al., 2017; Jing et al., 2018). The efficacy of these defense networks and their targets during desiccation and rehydration are well documented in plants (Hoekstra et al., 2001; Zhang and Bartels, 2018; Oliver et al., 2020). Furthermore, down-regulation of genes involved in the cell cycle, DNA processing, and primary metabolism free up more energy for resistance to dehydration stress (Buitink et al., 2006; Franchi et al., 2011; Maia et al., 2011; Oliver et al., 2020). With the arrest of growth, the production of reactive oxygen species (ROS) is decreased to a minimum, which minimizes damage to the cells.

These observations support the idea that physiological and molecular processes in seed DT seem to be able to provide suitable strategies for studying DT in seeds. However, the emergence of DT acquisition overlaps with seed maturation. Hence it is challenging to unequivocally separate genes conferred by the emergence of DT acquisition from those conferred by seed development. For example, the LAFL (LEAFY COTYLEDON, ABSCISIC ACID INSENSITIVE, FUSCA, and LEC) transcription factor network not only regulates seed development but also diverse seed-specific processes, including deposition of storage reserves (starch, storage proteins, and lipids), acquisition of DT, developmental arrest of the embryo, and dormancy (Jia et al., 2014). It is difficult to identify whether a transcription factor is involved in DT acquisition per se during seed development. In addition, genes associated with morphological changes of seeds during development also obstruct the screening of genes associated with DT. In addition, the experimental period of this system is determined by the period of seed development, which can be uncertain and time-consuming to characterize in full (**Table 1**).

#### DT loss during seed germination

It is well established that orthodox seeds lose their DT and become sensitive to extreme drying during the early stages of visible germination (Koster et al., 2003; Bewley et al., 2013). The loss of DT is correlated with the start of cell division soon after radicle emergence. The switch from DT to being desiccation-sensitive (DS) coincides with radicle cells containing double-strand DNA entering the G2 phase of the cell cycle (Saracco et al., 1995; Faria et al., 2005). In contrast to the seed developmental stage, many transcription factors (MYB, AP2, and NAC), and gene encoding methyltransferase and histone, are differentially expressed (Tian et al., 2016). And the DT relative genes such as LEA and HSP are down-regulated suggesting the loss of these proteins alters the DT of a germinating seedling (Mtwisha et al., 2007; Ginsawaeng et al., 2021).

Although using this system to explore the loss of DT generally shortens the experimental period, it has two limitations. Firstly, the physiological changes in seed germination would interfere DT evaluation. Secondly, this system is the reverse process of DT acquisition in orthodox seeds, being a loss of function rather than a gain of function process. Intriguingly, there is the potential to reverse the loss of DT as a result of the onset of visible germination. This physiological response provides a valuable means of dissecting the molecular, biochemical and temporal foundation of DT re-establishment in the germinating seed.

#### DT re-establishment in germinating seeds

Germinating orthodox seeds have a window to tolerate re-drying, which can be prolonged by applying mild osmotic stress (*e.g.*, using polyethylene glycol [PEG] solution) and/or using plant hormone (abscisic acid [ABA]) before relatively fast-drying is applied. DT has been successfully re-established for seeds of 12 species (**Table 2**), including those of herbaceous plants (*e.g., Arabidopsis thaliana*, *Cucumis sativus*, *Impatiens walleriana*, *Medicago truncatula, Pisum sativum*, *Vigna unguiculata*, and *Xerophyta viscosa*) and woody plants (*e.g., Cedrela fissilis*, *Caragana korshinskii*, *Peltophorum dubium*, *Sesbania virgata*, and *Tabebuia impetiginosa*) (Bruggink and van der Toorn, 1995; Buitink et al., 2000; Leprince et al., 2000; Buitink et al., 2003; Leprince et al., 2004; Faria et al., 2005; Avelange-Macherel et al., 2006; Boudet et al., 2006; Buitink et al., 2006; Vieira et al., 2010; Maia et al., 2011; Terrasson et al., 2013; Lyall et al., 2014; Maia et al., 2014; Masetto et al., 2014; Costa et al., 2015a; Costa et al., 2015b; Masetto et al., 2016; Costa et al., 2016; Guimarães et al., 2016; Lang et al., 2017; Peng et al., 2017; Silva et al., 2017; Peng et al., 2018; Peng et al., 2021; Wang et al., 2021).

**Table 2:** The studies of re-establishment DT in germinating seeds.

Species	Inductor	Concentration	Temperature	Duration of treatment	Reference
*Arabidopsis thaliana*	PEG	-2.5 MPa	22°C	3 d	Maia et al., 2011
	PEG	-2.5 MPa	22°C	3 d	Lang et al., 2017
	ABA	10 μM	20°C	3 d	Costa et al., 2015a
	ABA	5 μM	22°C	10 d	Maia et al., 2014
*Caragana korshinskii* Kom	PEG/H_2_O_2_	-1.7 Mpa/1 mM	5°C/25°C	3 d/radicles elongated to 2 mm	Peng et al., 2017
	PEG/ABA	-1.7 Mpa/1 mM	5°C/20°C	3 d/3 d	Peng et al., 2018
	PEG/ABA	-1.7 Mpa/1 mM	5°C/20°C	3 d/3 d	Peng et al., 2021
*Cedrela fissili*s Vell.	PEG+ABA	-2.04 MPa+100 μM	5°C	3 d	Masetto et al., 2014
*Cucumis sativa*	PEG	-1.5 Mpa	10°C	3 d	Avelange-Macherel et al., 2006
	PEG	-1.5 Mpa	10°C	7 d	Leprince et al., 2000
	PEG	-1.5 Mpa	8°C	6 d	Bruggink and van der Toorn, 1995
	PEG	-1.5 Mpa	10°C	3 d	Leprince et al., 2004
*Impatiens walleriana*	PEG	-1.5 Mpa	8°C	6 d	Bruggink and van der Toorn, 1995
*Medicago truncatula*	PEG	-1.7 Mpa	10°C	3 d	Leprince et al., 2004
	PEG	-1.7 Mpa	10°C	2 d	Boudet et al., 2006
	PEG	-1.7 Mpa	10°C	3 d	Terrasson et al., 2013
	PEG	-1.8 Mpa	5°C	3 d	Faria et al., 2005
	PEG	-1.7 Mpa	10°C	3 d	Buitink et al., 2006
	PEG	-1.7 Mpa	10°C	3 d	Buitink et al., 2003
*Pisum sativum* L.	PEG	-1 Mpa	10°C	2 d	Wang et al., 2021
*Peltophorum dubium*	ABA	5 µM	4°C	3 d	Guimarães et al., 2016
*Sesbania virgata (Cav.)* Pers.	PEG	-2.04 MPa	5°C	3 d	Masetto et al, 2016
	PEG	-2.5 MPa	4°C	3 d	Costa et al., 2016
*Tabebuia impetiginosa* Mart.	PEG	-1.7 Mpa	5°C	3 d	Vieira et al., 2010
*Vigna unguiculata*	PEG	-1.7 Mpa	5°C	1 d	Silva et al., 2017
*Xerophyta viscosa*	PEG/sucrose	-2.5 Mpa/3% (w/v)	22°C/22°C	2 d/Germinated to each developmental stage	Lyall et al., 2014

In the PEG or ABA-induced DT re-establishment system, germinating seeds return to the dormant or quiescent state (*i.e.* metabolically inactive) to exclude interference by seed developmental and germinative morphological alteration (Maia et al., 2011; Costa et al., 2015a; Dekkers et al., 2015; Peng et al., 2018). This technological process only takes approximately 2 weeks to apply as it comprises five steps (germination, PEG or ABA treatment, dehydration, pre-humidification, and rehydration) (**Figure 1**). Because there are well defined control points during this germination-based system, it is convenient for detailed studies on seed DT in a broad range of species, including xerophytes.

The DT re-establishment system is artificial processing rather than natural. Therefore, more experimental factors influence the efficiency and stability of the system. It may lead to the progress of re-establishment DT is not plain sailing (**Table 1**) (Maia et al., 2011; Peng et al., 2017).

### Progress in re-establishment of seed DT

Long after the germination process has been triggered during imbibition of non-dormant seeds, the seeds will completely lose their DT and irreversibly become sensitive to desiccation. Hence, DT re-establishment is heavily dependent on timing within the germinative window (Leprince et al., 2000; Buitink et al., 2003; Vieira et al., 2010; Maia et al., 2011). *A. thaliana* seeds can be triggered to re-establish DT, and four clearly distinct germination stages have been defined (Maia et al., 2011). In the first three stages, seeds are able to withstand desiccation after a PEG treatment (-2.5 MPa). However, this ability is largely lost at stage IV when the first root hair is visible (Maia et al., 2011; Maia et al., 2014). Similar to *A. thaliana* seeds, after PEG treatment (-1.7 MPa) most seeds of 2.7 mm long radicles in *M. truncatula* survived and developed into normal germinating seeds after rehydration (Buitink et al., 2003).

Another feature is that different seed parts display variable levels of DT (**Figure 1**). The radicles appearing to be the most desiccation-sensitive part of germinating seeds, followed by the hypocotyl and cotyledon tissues (Buitink et al., 2003; Maia et al., 2011; Peng et al., 2017). In *M. truncatula* and *C. korshinskii*, radicles as experimental materials used for DT re-establishment (Buitink et al., 2003; Peng et al., 2017). In addition to the tissue part of seeds, osmotic potential, temperature, and treatment time are factors that also influence the re-establishment of DT in germinating seeds at PEG concentrations varying from -1 to -2.5 MPa (Maia et al., 2011; Peng et al., 2017; Wang et al., 2021).

The availability of a robust system of modulating the re-establishment of physiological DT in seeds facilitates multi-omics studies on discovering genes and pathways linked to how seeds survive drying. Transcriptome profiling and proteomic analyses have uncovered key metabolic and regulatory processes during DT re-established by PEG treatment in *M. truncatula* (Buitink et al., 2003; Boudet et al., 2006) and *A. thaliana* seeds (Maia et al., 2011). Transcripts of germinating seeds after PEG treatment are dominated by those encoding LEA, seed storage proteins, and dormancy-related proteins (Buitink et al., 2003; Boudet et al., 2006; Maia et al., 2011). In parallel, there is massive repression of genes belonging to many other classes, such as those involved in photosynthesis, cell wall modification, and energy metabolism (Buitink et al., 2003; Boudet et al., 2006; Maia et al., 2011). It also appears that mainly starch and lipid reserves, and to a lesser extent oligosaccharides, are mobilized at different time points to provide sucrose for osmotic adjustment and protection (Buitink et al., 2003). Overall, these changes facilitate a programmed reversion from a metabolic active state to a quiescent state.

According to the gene expression profile, the up-regulated genes in PEG treated seeds are related to the acquisition of DT while the down-regulated genes are related to imbibition. The down-regulated genes in PEG treated seeds are up-regulated upon imbibition. It appears that germinating seeds after PEG treatment indeed revert to a developmental DT stage (Buitink et al., 2003; Maia et al., 2011). From a regulatory point of view, several transcription factors modulate the regulatory network relative to LEA, HSPs, and seed longevity. Of note, a marked enrichment of transcription factors to the promoters of the most highly up-regulated DT-associated genes has been reported (Maia et al., 2011; Terrasson et al., 2013).

Besides PEG treatment, DT in germinating seeds can also be rescued by treatment with ABA (Maia et al., 2014; Costa et al., 2015a). In agreement with the role of PEG, the main physiological and molecular processes that occur during incubation in ABA reveal growth arrest and a partial return to the quiescent stage (Maia et al., 2014; Costa et al., 2015a). During ABA-induced DT re-establishment, the expression of a large number of genes encoding abiotic stress response TFs, such as ABI5, APETALA 2/ethylene-responsive element- binding factor (AP2/ERF) family, NAM/ATF1/CUC2 (NAC) class and WRKY TFs, ABRE-binding factor 1 (ABF1), *etc*. are induced (Costa et al., 2015a). The re-establishment of DT depends on the modulation of ABA sensitivity rather than enhanced ABA content, emphasizing ABA receptors play a pivotal role in the re-establishment of DT in germinating seeds (Maia et al., 2014; Costa et al., 2015a).

It is evident that comprehensive omics data is shedding light on the molecular and physiological processes associated with the re-establishment of DT in seeds. However, the main experimental findings on the mechanisms of DT re-establishment in seeds are rather limited to the model plants *A. thaliana* and *M. truncatula*. Their seeds exhibit much weaker DT compared to xerophytic seeds and there are no unexpected genes or mechanisms that have been functionally characterized. Therefore, it is important to widen the search for functional DT re-establishment to species that grow in harsh environments, *e.g.*, xerophytic seeds with exceptional DT. In this way, it may be possible to identify new mechanisms of DT establishment in nature.

### DT re-establishment in germinating seeds of *caragana korshinskii* kom.

*Caragana korshinskii* Kom. is a perennial sandy grassland and desert deciduous shrub species with strong drought resistance, distributed in the northwest of China and Mongolia (Lai et al., 2015). Its seeds are non-dormant and germinate rapidly after imbibition (Abudureheman et al., 2014; Lai et al., 2015). Post-radicle emergence, DT decreased with mitosis in both *C. korshinskii* and *M. truncatula*, but the rate of DT loss during visible germination in *C. korshinskii* is significantly slower (Faria et al., 2005; Peng et al., 2017), *i.e.* the window is wider. These characteristics indicate that *C. korshinskii* can be a particularly suitable species to explore DT mechanisms.

The broad changes in the transcriptome in radicles of *C. korshinskii* show the complexity of the response of these seeds to PEG-induced re-establishment of DT, including the enhancement of many genes encoding stress-related proteins (HSPs and LEAs), raffinose family oligosaccharide biosynthesis-related enzymes (RFOs), and TFs (WRKY, NAC, and ERF) (Peng et al., 2017). In addition, ROS accumulates in the radicle tip of germinating *C. korshinskii* seeds during DT re-establishment. Given the dual function of ROS, the accurate execution of signaling functions of ROS prevents the accumulation of oxidative damage while enabling the re-establishment of DT in emerging radicles (Mittler, 2017; Peng et al., 2017).

In addition to PEG or ABA, hydrogen peroxide can act as an independent inducer of DT re-establishment (Peng et al., 2017). Although there are apparent similarities between the DT re-establishment following PEG or H_2_O_2_ treatments in *C. korshinskii*, a key difference exists regarding the phenylpropanoid–flavonoid pathway that avoid exogenous ROS damage. This pathway is activated by H_2_O_2_ treatment but is insensitive to PEG treatment (Peng et al., 2017). Consequently, these comprehensive mechanisms induced by hydrogen peroxide provide novel information on DT re-establishment, which is beneficial for the understanding the role of ROS in seed DT in seeds of xerophytic species and potentially other species.

In addition to the best-known proteins and genes relative to DT in the model plant, the proteomic analysis of *C. korshinskii* seeds showed the greatest increase in abundance of metallothionein (MT), a change not previously observed in other species (Peng et al., 2018; Peng et al., 2021). During seed DT re-establishment, in addition to metal-binding capacity, *in vivo* and *in vitro* evidences illustrate CkMT4 might supply Cu^2+^/Zn^2+^ to SOD under high redox potential provided by PEG treatment for excess ROS scavenging (Peng et al., 2018; Peng et al., 2021). *CkMT4* acts as a candidate gene for improving survival of germinating seeds under dehydration (Peng et al., 2021). Studies with *C. korshinskii* that identify putative key genes for adaptation may also help disentangle the evolutionary complexity of seed DT (Peng et al., 2021; Nonogaki et al., 2022).

## Discussion

The germinating seeds of *C. korshinskii* display stronger DT than model plants (Buitink et al., 2003; Peng et al., 2017). Building on the above findings, it appears that the xerophytic seeds represented by *C. korshinskii* have potential research value for studying DT. However, to our knowledge, little studies focus on DT of xerophytic seeds, except *C. korshinskii*. The research value and limited research of xerophytic seeds are out-off-balance. To bridge the gap, a future research program is necessary to explore the full potential of their stress tolerance. The DT re-establishment system provides a powerful tool for revealing the mechanisms of DT and for screening novel pivotal genes associated with DT (Peng et al., 2018; Peng et al., 2021). As a follow-up, we suggest programming the following steps to unveil the DT mechanism of xerophytic seeds and building the gene library related to DT.

Firstly, in addition to *C. korshinskii*, more xerophytic species should be applied to re-establish DT. Secondly, employ multi-omics methods to associate physiological processes with molecular mechanisms. Crosswise comparison of the DT re-establishment in numerous different species will reveal universal and unique regulation mechanism. Then seed scientists will screen the novel DT relative genes and decipher the functional characterization of these genes. In the last step, construct a gene library from the xerophytic plant to provide efficient candidate genes for improving DT in seeds.

Besides extensive research on DT in xerophytic seeds, the deciphering of the cellular or molecular process for *C. korshinskii* is just beginning and more subtle regulatory mechanic studies should be carried out. Below is a list of three selected questions that we believe require further investigation:

- What is the role of post-translational modification in the re-establishment of DT? Post-translational modification, as the frontier of signaling pathway regulatory studies, has been intensely studied in botany-related fields, especially plant stress resistance (Xu and Xue, 2019; Ding et al., 2020; Chen et al., 2021). During DT re-establishment in germinating seeds of *C. korshinskii, *the genes related to the degradation of the ubiquitin pathway exhibit a higher level of expression, which indicates ubiquitination may be involved in this process (Peng et al., 2017). We believe that this case also requires methylation, acetylation or phosphorylation, *etc*. Therefore, extensive epigenetic studies are needed to identify the molecule modification and explain their functionalities.
- Can the control of cell division under mild osmotic stress be optimized in relation to critical thresholds for germination (Maleki et al., 2022)? Plant growth and resistance involve a balance between metabolites and energy distribution relating to cell expansion and division. During DT re-establishment, seeds abandon the germination process for enhanced resilience (Maia et al., 2014; Costa et al., 2015a; Peng et al., 2018). Under this scenario, the direction of energetically expensive processes undergoes a sharp change. A switch should be presented that drastically alters the seeds’ behavior at particularly water potentials whereby the outcome of the physiological stage can be directed from germinal to quiescent. Considering the intimate connection between inhibition of cell division and the quiescent phase, it is obvious that the expression and regulatory networks of genes related to cell division need to be further analyzed.
- How to maintain the healthy state of DNA on the process of DT re-establishment in germinating seeds? In the process of DT re-establishment, PEG treatment alleviates DNA damage induced by dehydration (Faria et al., 2005). The ROS scavenging system may prevent DNA from oxidative damage (Leprince et al., 2000; Buitink et al., 2006; Maia et al., 2011; Lang et al., 2017; Peng et al., 2017; Peng et al., 2018; Peng et al., 2021). In seeds with DT, some DNA damage that occurs during dehydration or dry storage may be repaired (Osborne, 2000). We thus provided a hypothesis that DNA protection and DNA repair systems could be activated during DT re-establishment. Evolutionary processes gave rise to DNA repair tools that are efficient in repairing damaged DNA (Britt, 1999; Que et al., 2019). Nevertheless, little work has been done to identify how DNA repair systems reduce the degree of DNA degradation during DT re-establishment. It will be interesting to focus on dissecting DNA protection and repair pathways in DT re-establishment.

In conclusion, this article aims to direct more attention towards the value of scientific research using xerophytic seeds and focusing on their underlying genes that permit the DT. We estimate that many efficient genes will be identified by disentangling the physiologic process of DT in xerophytic seeds, providing candidate genes for improving DT in intermediate and recalcitrant seeds through genetic engineering, which may be beneficial for germplasm conservation. The re-establishment of DT in diversified xerophytic seeds will assist in developing the comprehensive exploration of DT. This challenging program will require more researchers to devote themselves to analyzing the mechanism of DT in xerophytic seeds in order to accelerate the process of DT seed breeding.

## Author contributions

LP drafted the manuscript, **Figure 1** and **Tables 1**, **2**. HX and HWP edited them. All authors contributed to the article and approved the submitted version.

## Funding

This work was supported by the National Natural Science Foundation of China (31971646), and the Fundamental Research Funds for the Central Non-profit Research of Chinese Academy of Forestry (CAFYBB2020QB002).

## Conflict of interest

The authors declare that the research was conducted in the absence of any commercial or financial relationships that could be construed as a potential conflict of interest.

## Publisher’s note

All claims expressed in this article are solely those of the authors and do not necessarily represent those of their affiliated organizations, or those of the publisher, the editors and the reviewers. Any product that may be evaluated in this article, or claim that may be made by its manufacturer, is not guaranteed or endorsed by the publisher.
